# Infection Temperature Affects the Phenotype and Function of Chimeric Antigen Receptor T Cells Produced *via* Lentiviral Technology

**DOI:** 10.3389/fimmu.2021.638907

**Published:** 2021-04-19

**Authors:** Xin Jin, Wenyi Lu, Meng Zhang, Xia Xiong, Rui Sun, Yunxiong Wei, Xiaoyuan He, Mingfeng Zhao

**Affiliations:** ^1^ Nankai University School of Medicine, Tianjin, China; ^2^ Department of Hematology, Tianjin First Central Hospital, Tianjin, China; ^3^ The First Central Clinical College of Tianjin Medical University, Tianjin, China

**Keywords:** Lentivirus, Infection temperature, chimeric antigen receptor (CAR), naive T cells, immune checkpoints

## Abstract

Chimeric antigen receptor (CAR)-T cell therapy has become an important method for the treatment of hematological tumors. Lentiviruses are commonly used gene transfer vectors for preparing CAR-T cells, and the conditions for preparing CAR-T cells vary greatly. This study reported for the first time the influence of differences in infection temperature on the phenotype and function of produced CAR-T cells. Our results show that infection at 4 degrees produces the highest CAR-positive rate of T cells, infection at 37 degrees produces the fastest proliferation in CAR-T cells, and infection at 32 degrees produces CAR-T cells with the greatest proportion of naive cells and the lowest expression of immune checkpoints. Therefore, infection at 32 degrees is recommended to prepare CAR-T cells. CAR-T cells derived from infection at 32 degrees seem to have a balance between function and phenotype. Importantly, they have increased oncolytic ability. This research will help optimize the generation of CAR-T cells and improve the quality of CAR-T cell products.

## Introduction

Chimeric antigen receptor (CAR)-T cell therapy is a kind of adoptive immunotherapy that uses genetic engineering to express one or more specific chimeric antigen receptors (CARs) on T cells (1–3). CARs are artificial fusion proteins consisting of an antigen-recognition region connected to a signal element through a hinge and a transmembrane region (4). Antigen recognition regions are usually single-chain variable fragments (scFvs) derived from antibodies. The signal domain includes the costimulatory domain from proteins such as CD28 and 4-1BB, and the activation domain is usually from CD3ζ. CAR-T cells can perform non-MHC-dependent antigen recognition to effectively bypass the tumor’s main immune escape mechanism, the downregulation of MHC molecules, thereby specifically killing tumor cells.

The introduction of CAR-T cell immunotherapy has been a milestone in tumor immunotherapy in recent years, especially in the immunotherapy of hematological tumors (5–7). CAR-T cells targeting CD19 and BCMA have shown significant antitumor effects in the treatment of relapsed/refractory (r/r) B cell tumors and multiple myeloma. Four anti-CD19 CAR-T cell products (tisagenlecleucel, axicabtagene ciloleucel, brexucabtagene autoleucel and breyanzi) have been approved by the FDA as drugs since 2017 (5). One anti-BCMA CAR T-cell product (idecabtagene vicleucel, bb2121, ide-cel) is likely to be approved by FDA (6, 7).

Virus-mediated gene transfer is widely used in the preparation of CAR-T cells because viral vectors can effectively transfer genes to a variety of cell types and can stably integrate into their genomes, leading to long-term gene expression, which persists in progeny cells (8). However, the infection conditions for preparing CAR-T cells from viruses vary greatly (9–20). Tisagenlecleucel uses a 37-degree infection (16), breyanzi and axicabtagene ciloleucel use a 32-degree infection (15, 21), and some studies have used room temperature or a 4-degree infection to produce CAR-T cells with viral vectors (12, 13, 17, 20). As seen above, temperature is a very important infection condition. Studies with retroviruses have shown that because the virus has a longer half-life at 32 degrees, 32 degrees is more efficient than 37 degrees for infecting cells (14, 18). Other studies have shown that the culture temperature has an impact on the productivity of mammalian cells and the stability of the virus (22–24). However, the optimal infection temperature in lentiviruses, which are widely used for gene delivery in the preparation of CAR-T cells, has not yet been studied. Here, we used different temperatures to observe the influence of the temperature used during lentiviral infection of T cells on the preparation of CAR-T cells; these experiments will help to determine the optimal infection temperature to improve the quality of CAR-T cell products.

## Materials and Method

### Cell Lines and Primary Cells

HEK-293T (ATCC) cells were grown in Dulbecco’s modified Eagle medium (DMEM; Gibco) supplemented with 10% fetal calf serum (FCS; Biological Industries). NALM-6 [peripheral blood B cell precursor leukemia cells (acute lymphocytic leukemia (ALL)), CD19+] and MOLM-13 [human acute myeloid leukemia cells (AML), CD123+] cells were grown in RPMI 1640 medium supplemented with 10% FCS (Gibco). All cell lines were cultured at 37 degrees, 5% CO2 and 95% humidity for up to 1 month. The cells were divided every 2 to 3 days, and the number of passages did not exceed 20. Peripheral blood samples were obtained from healthy donors (n = 3) in The Tianjin First Central Hospital after informed consent was obtained according to the institutional guidelines. Peripheral blood mononuclear cells (PBMNCs) were enriched through a Ficoll Hypaque gradient.

### Transgene Constructs

The scFv targeting CD19 originated from the FMC63 clone. The scFv targeting CD123 originated from the 7G3 clone. The CAR vectors contained the scFv and human 4-1BB and CD3ζ signaling domains, which were subcloned into the pCDH-MND-MCS-T2A-Puro lentiviral plasmid vector. The CAR sequence was preceded by the RQR8 tag separated by a short T2A peptide for detection (25).

### Lentivirus Production

The preparation of the lentivirus was performed according to the manufacturer’s instructions (GeneCopoeia). Briefly, two days before transfection, plate HEK-293T lentiviral packaging cells in a 10-cm dish in 10 ml of DMEM supplemented with 10% heat-inactivated fetal bovine serum so that the cells are 70–80% confluent at the moment of transfection. In a sterile polypropylene tube, dilute 2.5 µg of lentiviral expression plasmid and 5.0 µl (0.5 µg/µl) of Lenti-Pac HIV mix into 200 µl of Opti-MEM^®^ I (Invitrogen). In a separate tube, dilute 15 µl of EndoFectin Lenti into 200 µl of Opti-MEM I. Add diluted EndoFectin Lenti reagent drop-wise to the DNA solution while gently vortexing the DNA-containing tube. Incubate the mixture for 10–25 minutes at room temperature to allow the DNA-EndoFectin complex to form. Add the complex directly to each dish. Replace the overnight culture medium with fresh DMEM medium supplemented with 2–5% heat-inactivated fetal bovine serum. Add 1/500 volume of the TiterBoost reagent to the culture medium. Collect the pseudovirus-containing culture medium in sterile capped tubes 48 hours post transfection and centrifuge the tubes at 500g for 10 minutes to get rid of cell debris. Following centrifugation, filter the supernatant through 0.45 µm polyethersulfone low protein-binding filters. Viral supernatants were concentrated using ultracentrifugation at 50,000 for 2 hr 30 min. Virus-containing pellets were resuspended in complete X-Vivo15 media and stored at −80°C until use.

### Lentivirus Titration

The number of transducing units (TU/mL) was determined by the limiting dilution method. Briefly, HEK-293T cells were seeded 12 hr before transduction. Then, 1:10 dilutions of the viral supernatant were prepared and added on top of the cells in complete DMEM + 5 μg/mL Polybrene. Cells were trypsinyzed 72 hr later and labeled with the QBEND-10 monoclonal CD34 antibody (Abcam) before being analyzed by flow cytometry. A dilution corresponding to 2%–20% of positive cells was used to calculate viral titer.

### Production of CAR-T Cells

CD3+ T cells were separated from PBMCs using CD3 immunomagnetic beads (#130-097-043, Miltenyi Biotec, Germany) on day 1. T cells were amplified using CD3/CD28 stimulation beads (#11131D, Thermo Fisher Scientific) and IL-2(100 IU/mL; Miltenyi Biotec) in X-VIVO 15 Cell Medium (Lonza). Cells were then activated and expanded for 48 hours were transduced 2 hr later with the lentivirus (multiplicity of infection is 10) by different temperatures incubation in the presence of polybrene (Sigma) at 8 μg/mL. Then, the cells continue to expand at 37 degrees at an appropriate concentration (0.5-1×106 cells/ml). The transduction efficiency was determined 3 days after transduction. Generally, the T cells were engineered *via* 9-12 days of manufacturing to express a CD19-specific CAR or CD123-specific CAR.

### Detection of CAR Expression by Transduced T Cells

For each analyzed T cell culture, one sample of cells was stained with Alexa-Fluor 647-labeled polyclonal goat anti-mouse IgG (H+L) antibodies (Affinity) to detect CAR-T cells. In addition, we also detected the expression of the CAR with the QBEND-10 monoclonal CD34 antibody (Abcam) labeled with an RQR8 tag. Subsequently, all cells were stained with fluorescein isothiocyanate (FITC)-labeled anti-CD3 antibodies (Abcam).

### Immunophenotyping

Anti-human monoclonal antibodies against CD3 (Biolegend), CD4 (Biolegend), CD8 (Biolegend), CD34 (Abcam), CD45RO (Biolegend), CD62L (Biolegend), PD1 (Biolegend), LAG3 (Biolegend), and TIM3 (Biolegend) were used for immunophenotypic analysis. All flow cytometry analyses of stained cells were performed with a Coulter Altra flow cytometer equipped with CytExpert software (Beckman Coulter).

### Assessment of Cytokines

Toxicities were evaluated relative to a baseline assessment conducted before CAR-T cell infusion. The concentrations of serum inflammatory markers, including IL-2, IL-4, IL-6, IL-10, TNF-α, IFN-γ and GM-CSF were evaluated by Luminex assay according to the manufacturer’s instructions.

### Cytotoxicity Determination

CD19+ NALM-6 cells and CD123+ MOLM-13 cell lines were used to determine the cytotoxic activity of CD19 CAR-T and CD123 CAR-T cells, respectively. CAR expression was detected 72 hours after transduction by flow cytometry, and CAR-T cell cytotoxic activity was evaluated the next day. NALM-6 CAR-T cells were previously labeled with CellTrace CFSE (Invitrogen) according to the manufacturer’s instructions. To compensate for the change in transduction efficiency, the effector cell population was normalized to the absolute number of T cells by adding untransduced T cells. NALM-6 cells without effector cells were used as a control. After 24 hours of incubation, the cell mixture was stained to visualize dead cells using the fixable viability dye eFluor 780 (Thermo Fisher Scientific) according to the manufacturer’s instructions and analyzed by flow cytometry. The percentage of dead target cells was determined using the CFSE-positive and viability dye-positive cell population (19).

### *In Vivo* Leukemia Xenograft Study

Male NSG mice (Sipeifu) aged 5-6 weeks were injected intravenously with 2×10^6^ luciferase-expressing NALM-6 cells cultured in our laboratory. Three days later, 5×10^6^ CD19 CAR-T cells or uninfected T cells were injected into the mice through the tail vein. To monitor tumor growth, each mouse was injected intraperitoneally with 3 mg of D-luciferin (Sigma, US) at the designated time point, and the mice were imaged using an IVIS Imager 10 minutes later.

### Statistical Analysis

The results were analyzed with the GraphPad Prism program (GraphPad Software). Data that obeyed a normal distribution are presented as the mean ± standard deviation (SD), and multiple group comparisons were performed by using one-way analysis of variance (ANOVA), whereas data with a nonnormal distribution are shown as the median and quartiles and were compared by the Kruskal-Wallis test. The survival curves were analyzed using the Kaplan-Meier method with the log-rank test. P<0.05 was considered statistically significant.

## Result

### Successful Preparation of CAR-T Cells

**Figure 1:**
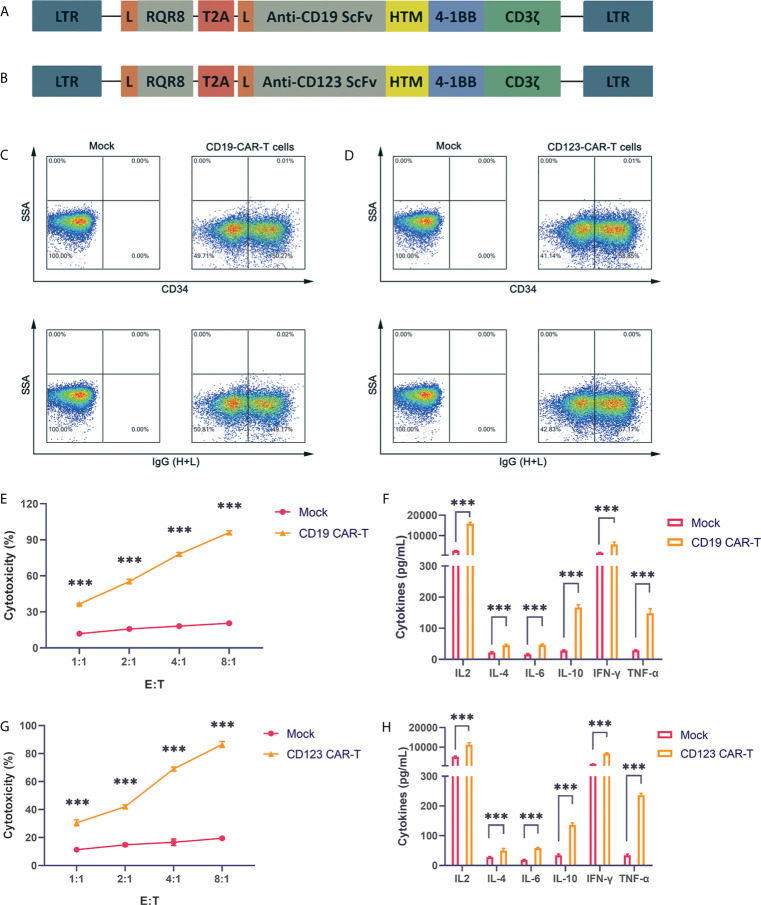
Successful production of well-functioning CAR-T cells **(A)** CD19 CAR vector schematic diagram. HTM is the CD8 hinge and transmembrane region. **(B)** CD123 CAR vector schematic diagram. **(C)** Anti-CD34 antibody and anti-IgG (H+L) antibody labeling of CAR-T cells, and detection of the infection rate of CD19 CAR-T cells by flow cytometry. **(D)** Anti-CD34 antibody and anti-IgG (H+L) antibody labeling of CAR-T cells, and detection of the infection efficiency of CD123 CAR-T cells by flow cytometry. **(E)** Cytotoxicity of CD19 CAR-T cells and CD19-positive NALM-6 cells coincubated at different effector:target (E:T) ratios for 24 hours. **(F)** Coincubation of CD19 CAR-T cells and CD19-positive NALM-6 cells at a 1:1 ratio, and detection of the secretion levels of IL-2, IL-4, IL-6, IL-10, IFN-γ and TNFα in the supernatant. **(G)** Cytotoxicity of CD123 CAR-T cells and CD123-positive MOLM-13 cells coincubated at different E:T ratios for 24 hours. **(H)** Coincubation of CD123 CAR-T cells and CD123-positive MOLM-13 cells at a 1:1 ratio, and detection of the secretion levels of IL-2, IL-4, IL-6, IL-10, IFN-γ and TNFα in the supernatant. Three independent experiments were conducted. Mean ± SD. ***p < 0.001.

CD19 is expressed on the surface of B cell tumors. The three CAR-T cell drugs currently on the market all target CD19. Therefore, research on CD19 CAR-T cells is very important. Our CAR structure contains the FMC63 single-chain antibody, the CD8 hinge region and transmembrane region, the 41-BB costimulatory signal and the CD3ζ signal domain. The structure is similar to the structures of Novartis products (**Figure 1A**). To obtain more reliable results, we also constructed a CAR vector targeting the CD123 molecule (**Figure 1B**). CD123 is mainly expressed on the surface of myeloid cells and targets AML cells. We also added the RQR8 tag sequence to detect the expression of the CAR. The tag can be labeled with the QBEND-10 monoclonal CD34 antibody. Through lentivirus-mediated gene transfer, the CAR fusion protein was successfully expressed on the surface of activated T cells, and CD19 CAR-T and CD123 CAR-T cells with high infection rates were obtained (**Figures 1C, D**). The produced CD19 CAR-T cells and CD19-positive NALM-6 cells were coincubated at different ratios. Compared with uninfected T cells, CD19 CAR-T cells could significantly lyse NALM-6 cells (**Figure 1E**). Detecting the cytokines in the supernatant showed that the levels of IFN-γ, IL-6, IL-2, TNFα and other cytokines in the culture medium after CD19 CAR-T cells and target cells were coincubated were significantly increased (**Figure 1F**). We coincubated CD123 CAR-T cells with the CD123-positive MOLM-13 AML cell line, detected cytotoxicity and cytokines, and obtained similar results to those for the CD19 CAR-T cells (**Figures 1G, H**). The above data prove that our CAR-T cells targeting CD19 and CD123 were successfully produced, and these CAR-T cells could kill specific target cells and secrete abundant cytokines.

### Infection Temperature Can Affect the Proliferation and Infection Efficiency of Produced CAR-T Cells

**Figure 2:**
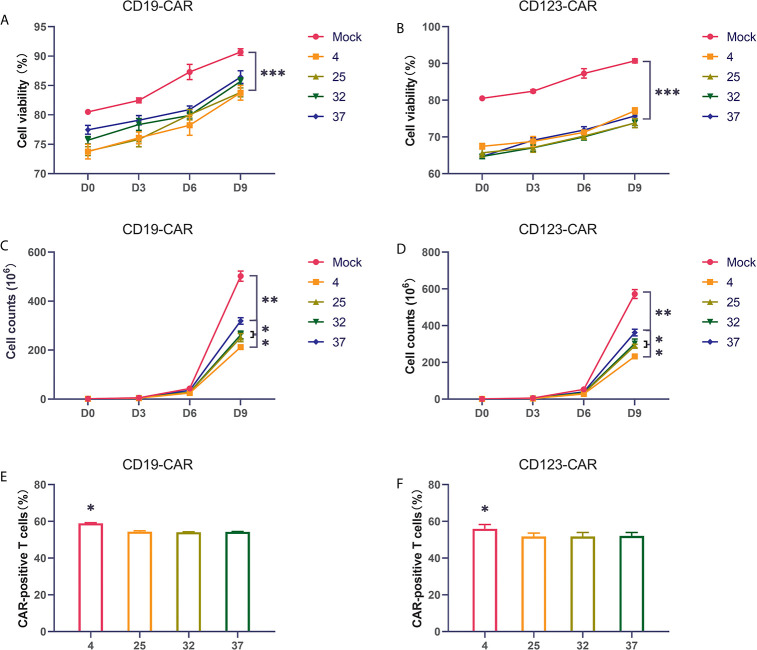
Infection temperature can affect the proliferation and infection efficiency of CAR-T cells **(A, B)** The proportion of living CD19 and CD123 CAR-T cells produced at different infection temperatures on the first day, the third day, the sixth day, and the ninth day after infection. **(C, D)** The number of living CD19 and CD123 CAR-T cells produced at different infection temperatures on the first day, the third day, the sixth day, and the ninth day after infection. **(E, F)** The infection efficiency of CD19 and CD123 CAR-T cells produced at different infection temperatures. Three independent experiments were conducted. Mean ± SD. *p < 0.05, **p < 0.01, ***p < 0.001.

Many studies have found that the temperature during virus infection affects the activity and proliferation of host cells. To explore the impact on CAR-T cells, we selected several commonly used temperatures in previous studies, namely, 4 degrees, 25 degrees, 32 degrees, and 37 degrees, for our experiments. Two hours after T cells were activated by lentivirus infection, they were placed in an incubator for normal culture, and the cells were counted on the 3rd, 6th, and 9th days of culture. Compared with uninfected T cells, CD19 CAR-T cells and CD123 CAR-T cells infected with virus had obviously lower cell viability and proliferation rates (**Figures 2A–D**). The proliferation rate of the 37-degree-infection group was significantly higher than the proliferation rates of the infection groups employing other temperatures, and the 4-degree-infection group had the lowest proliferation rate. Compared with uninfected T cells, CAR-T cells have lower cell activity. There is no significant difference in cell viability among the other temperature groups. Infection efficiency is a very important point in the preparation of CAR-T cells. High infection efficiency can save costs and time for cell culture *in vitro* and improve the success rate of treatment. We used flow cytometry to detect the CD19 CAR-T cell infection efficiency. Our results showed that the proportion of CAR-positive T cells in the 4-degree-infection group was higher than those in the other groups, which showed similar proportions (**Figure 2E**). The same result was also verified in CD123 CAR-T cells (**Figure 2F**). In addition, we also detected the secretion of cytokines in the culture medium on the 6th day of culture. Compared with that of uninfected T cells, CD19 CAR-T cell culture media has significantly higher levels of secreted cytokines. There was no significant difference in cytokine levels among the other temperature groups (**Supplement 1A–F**).

### Infection Temperature Can Affect CAR-T Cell Phenotype

**Figure 3:**
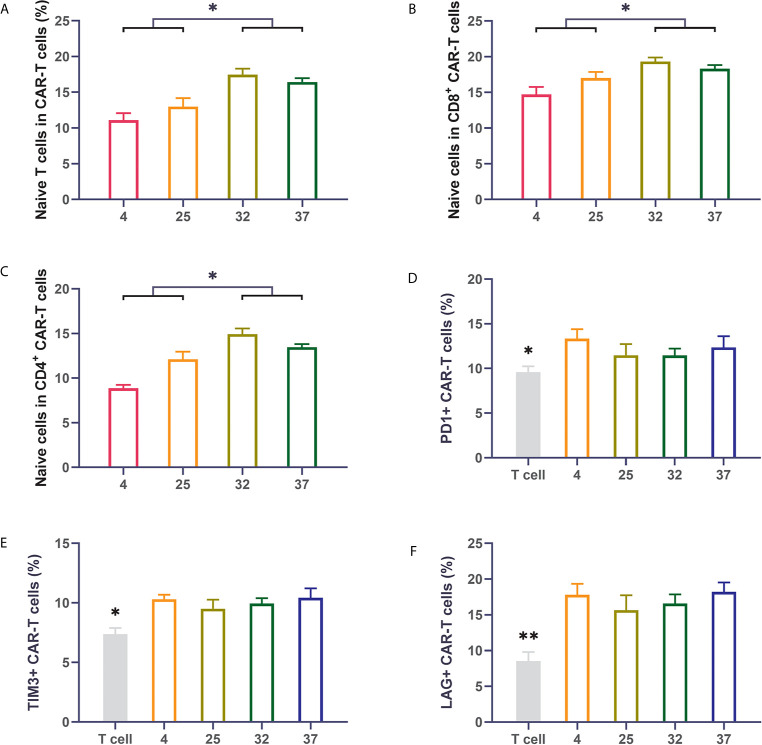
Infection temperature can affect the proportions of CD19 CAR-T cell subsets and the expression of immune checkpoints **(A–C)** The proportion of naive T cells among CD3-positive, CD8-positive and CD4-positive CD19 CAR-T cells generated at different infection temperatures. **(D–F)** The expression of PD1, TIM3, and LAG3 on the surface of CD19 CAR-T cells generated at different infection temperatures. Three independent experiments were conducted. Mean ± SD. *p < 0.05, **p < 0.01.

Previous reports have shown that the subpopulation distribution of CAR-T cells can impact the therapeutic effect of CAR-T cells. CAR-T cell products with a high proportion of naive T cells have better therapeutic effects after infusion than those with a low proportion of naïve T cells (26–28). We used flow cytometry to detect the proportion of naive cells (CD62L+CD45RO-) in the CAR-T cells. Compared with uninfected T cells, T cells infected with virus had an obviously lower proportion of naive T cells. The 32-degree- and 37-degree-infection groups had higher proportions of naive T cells, in terms of both CD8-positive T cells or CD4-positive cells, than the other infection groups (**Figures 3A–C**). Immune checkpoint expression is an important indicator for evaluating the quality of CAR-T cell products. CAR-T cells with lower expression of immune checkpoints have better therapeutic effects than those with higher expression of immune checkpoints. We detected the expression of PD1, TIM3 and LAG3 on the surface of CAR-T cells (29–32). Our results showed that compared with uninfected T cells, T cells infected with virus had a significantly higher percentage of immune checkpoint expression. There was no significant difference in the expression of immune checkpoints between different temperature groups, but a lower expression trend was observed in the 23-degree group and the 32-degree group (**Figures 3D–F**). These results were also reproduced in CD123 CAR-T cells (**Supplement 2A–F**).

### 32-Degree Infection Generates CAR-T Cells With the Best Killing Activity and Cytokine Secretion

**Figure 4:**
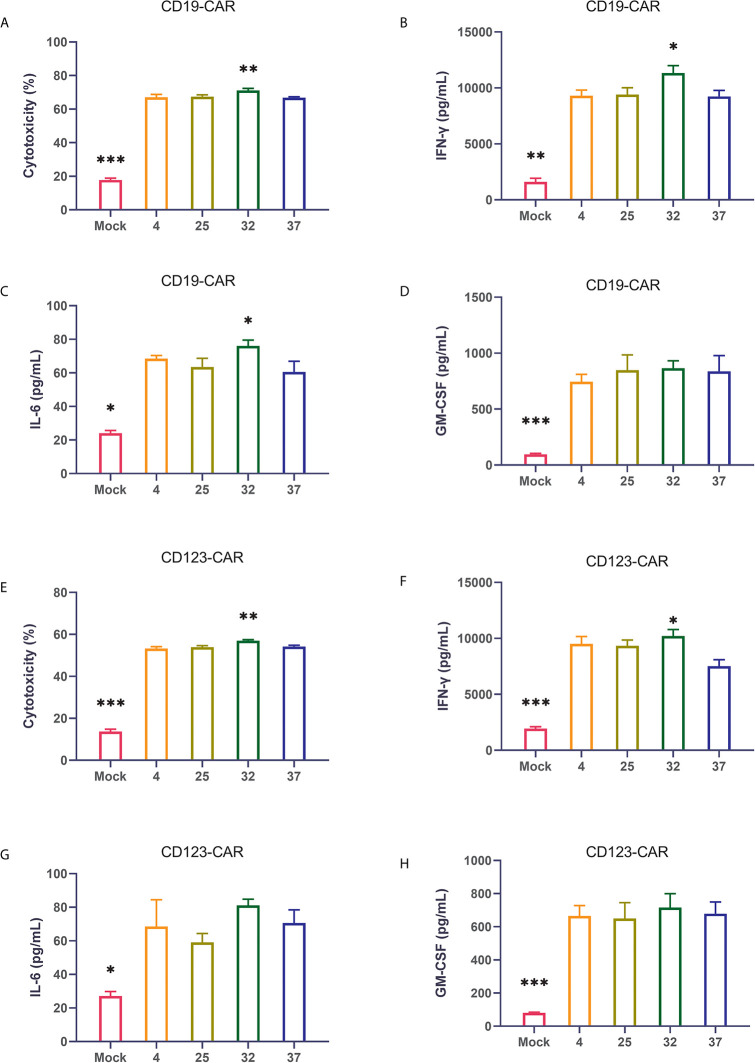
32-Degree infection generates CAR-T cells with optimal cytotoxicity and cytokine secretion **(A–D)** Determination of the cytotoxicity of CD19 CAR-T cells generated at different infection temperatures. CD19 CAR-T cells were coincubated with CD19-positive NALM-6 cells for 24 hours, and the concentrations of IFN-γ, IL-6 and GM-CSF in the culture supernatant were detected. **(E–H)** Determination of the cytotoxicity of CD123 CAR-T cells generated at different infection temperatures. CD19 CAR-T cells were coincubated with CD123-positive MOLM-13 cells for 24 hours, and the concentrations of IFN-γ, IL-6 and GM-CSF in the culture supernatant were detected. Three independent experiments were conducted. Mean ± SD. *p < 0.05, **p< 0.01, ***p < 0.001.

The ultimate function of CAR-T cells lies in their oncolytic ability. Therefore, we coincubated CAR-T cells from different temperature groups and target tumor cells at a ratio of 1:1 and then used flow cytometry to detect cytotoxicity. Compared with uninfected T cells, all CAR-T cells killed tumor cells significantly. The results showed that the CAR-T cells infected at 32 degrees had the strongest killing activity of the CAR-T cell groups infected at the 4 different temperatures (**Figure 4A**). The same result was also verified in CD123 CAR-T cells (**Figure 4E**). Cytokine storm is one of the problems faced by CAR-T cell therapy. To detect the ability of the CAR-T cells to induce cytokine release syndrome (CRS)-related toxicity, we used flow cytometry to detect the expression levels of related cytokines. Our results showed that CD19 CAR-T cells had significantly higher levels of cytokine secretion than uninfected T cells. Compared with the CAR-T cells generated at other temperatures, the CAR-T cells infected at 32 degrees secreted more cytokines, especially IFN-γ (**Figures 4B–D** and **Supplement 3A–D**). The same result was also verified in CD123 CAR-T cells (**Figures 4F–H** and **Supplement 3E–H**).

### The Effect of Infection Temperature on CAR-T Cells Is Not Clearly Reflected in the Mouse Tumor Model

**Figure 5:**
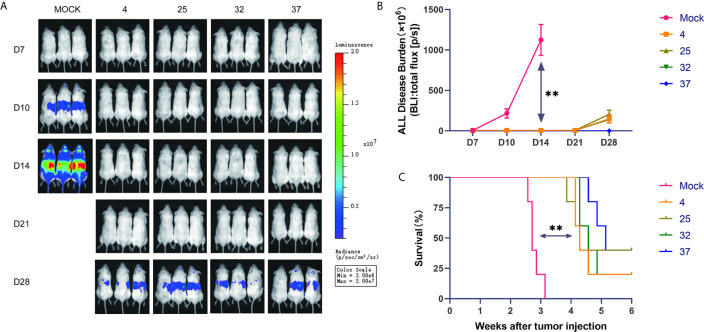
Effect of infection temperature on CAR-T cells in a mouse tumor model **(A)** Mice were injected with luciferase-containing NALM-6 cells through the tail vein. On the third day, the prepared CAR-T cells were injected into the mice through the tail vein. The tumor load in the mice was detected by *in vivo* imaging technology on the 7th, 10th, 14th, 21st and 28th days. (n=5, each group gives a representative picture of 3 mice). **(B)** Quantitative graphs of tumor burden in each group of mice at different time points are shown. **(C)** Survival of mice injected with uninfected T cells or CD19 CAR-T cells generated at different infection temperatures. Mean ± SD. **p < 0.01.

To further verify the results of our *in vitro* experiments, we injected mice with luciferase-expressing NALM-6 cells through the tail vein. On the third day after the tumor cells were injected, we injected the produced CAR-T cells into the mice through the tail vein. In vivo imaging technology was used to detect tumor burden in mice at specific time points. Our results show that compared with the uninfected T cell group, the group of mice injected with CAR-T cells had a significantly lower tumor burden and longer survival time (**Figures 5A–C**). The lack of obvious differences between mice injected with CAR-T cells from each temperature group may be due to the considerable heterogeneity between mice.

## Discussion

Recently, CAR-T cell therapy has become an important method for the treatment of hematological malignancies. There are a variety of methods available for gene delivery in CAR-T cell preparation, including lentivirus, retrovirus, mRNA, transposon, and sleeping beauty methods. Lentiviruses have a wide host range, infect both dividing and nondividing cells, and can persistently express the delivered genes. Therefore, lentiviruses are widely used as gene transfer vectors in the production of CAR-T cells. The conditions under which the lentivirus infect T cells in the production of CAR-T cells are a key factor. Here, we studied the effects of different infection temperatures on the preparation of CAR-T cells. Our results recommend using 32-degree infection because the CAR-T cells produced by 32-degree infection seem to have a balance between function and phenotype.

To confirm our results, we constructed two CAR structures that target different antigens. First, we successfully produced CAR-T cells by infecting activated T cells with lentivirus. Cell function verification was then carried out, confirming that both types of CAR-T cells could lyse tumor cells and secrete sufficient cytokines. It is worth noting that our CD19 CAR gene sequence was similar to the sequence used in tisagenlecleucel (16), which was approved by the FDA in 2017, suggesting that our CD19 CAR-T cell may be suitable for practical applications.

Cell viability and proliferation capacity are important quality parameters for CAR-T cell products. Our test results show that cells infected at 37 degrees had the highest proliferation capacity, which may be because the 37-degree environment is more conducive to cell growth. However, CAR-T cells infected at 4 degrees had the highest infection efficiency, which may be related to the fact that a low temperature is beneficial to the survival of the virus.

The proportions of cell subsets within CAR-T cell populations and checkpoint expression levels have now become important indicators for measuring the function of CAR-T cells. Previous studies have confirmed that a large proportion of naive T cells and low expression of immune checkpoints are related to improved prognosis of CAR-T cell therapy (26–32). We used flow cytometry to detect the distribution of cell subsets within CAR-T cells and checkpoint expression levels. In the 32-degree- and 37-degree-infection groups, the proportions of naive cells were significantly higher than those in the 4-degree- and 23-degree-infection groups. The checkpoint expression level in the 25-degree- and 32-degree-infection groups was relatively low, but there was no statistical difference. The reason why the 4-degree-infection group had the lowest proportion of naive cells remains to be further explored. Next, we further verified the functional differences of CAR-T cells generated at different infection temperatures through cytotoxicity and cytokine secretion analyses. Although CAR-T cells generated at 4 degrees had a higher infection rate than CAR-T cells generated at different temperatures, they did not show the strongest cytotoxicity or secretion of cytokines when they were incubated with target cells *in vitro*. This may be related to the higher expression of immune checkpoints and the lower proportion of naive cells in the CAR-T generated at degrees. In addition, the CAR-T cells generated at 32 degrees had the strongest cytotoxicity and greater cytokine secretion than those generated at 4 degrees. Later, we conducted an *in vivo* model study, and the survival time and *in vivo* tumor burden of mice injected with CAR-T cells from different infection temperature groups were not significantly different. Reducing the heterogeneity in tumor burden in the mouse model and expanding the mouse sample size may help to detect differences.

Novartis and Bristol Myers use lentiviruses to infect at 37 degrees and 32 degrees respectively to produce CAR-T cells (16, 21), and Kite Pharma uses a 32-degree infection with gamma-retrovirus to infect T cells (15). This article only studied the infection temperature used for lentiviruses. Whether the optimal temperature for T cell infection is similar between γ-retroviruses and lentiviruses, which belong to the Retroviridae family, needs further research. Development of semi-automated devices that can reduce the hands-on time and standardize the production of clinical-grade CAR T-cells, such as CliniMACS Prodigy from Miltenyi, is key to facilitate the development of CAR T-cell therapies (33, 34). Our research is done in well plates or culture flasks. Whether this view is consistent in semi-automatic equipment remains to be studied. In summary, our results report for the first time that temperature can affect the function and phenotype of CAR-T cells produced by lentivirus infection. We recommend using lentivirus to infect T cells at 32 degrees to produce CAR T cells. This research may provide an important reference for the production of CAR-T cell products.

## Data Availability Statement

The raw data supporting the conclusions of this article will be made available by the authors, without undue reservation.

## Ethics Statement

The studies involving human participants were reviewed and approved by Ethics Committee of Tianjin First Central Hospital. The patients/participants provided their written informed consent to participate in this study. The animal study was reviewed and approved by Ethics Committee of Tianjin First Central Hospital.

## Author Contributions

MFZ designed the research. XJ, WYL, MZ, XX, RS, and YXW performed the research. XJ and MFZ analyzed the data. XJ, WYL, MZ, and MFZ wrote the manuscript. XJ, WYL, and MFZ revised the manuscript. All authors contributed to the article and approved the submitted version.

## Funding

This work was supported by grants from the General Project of National Natural Science Foundation of China (81970180 to MZ), and the Key Science and Technology Support Project of Tianjin Science and Technology Bureau (20YFZCSY00800 to MZ), as well as Tianjin First Central Hospital.

## Conflict of Interest

The authors declare that the research was conducted in the absence of any commercial or financial relationships that could be construed as a potential conflict of interest.
