# Supplementary material for: Infection Temperature Affects the Phenotype and Function of Chimeric Antigen Receptor T Cells Produced via Lentiviral Technology
Source: Front Immunol. 2021 Apr 19;12:638907. doi: 10.3389/fimmu.2021.638907 (PMC8089475; doi:10.3389/fimmu.2021.638907)
Supplement: Supplementary file 1 [file DataSheet_1.pdf]

Supplementary Material

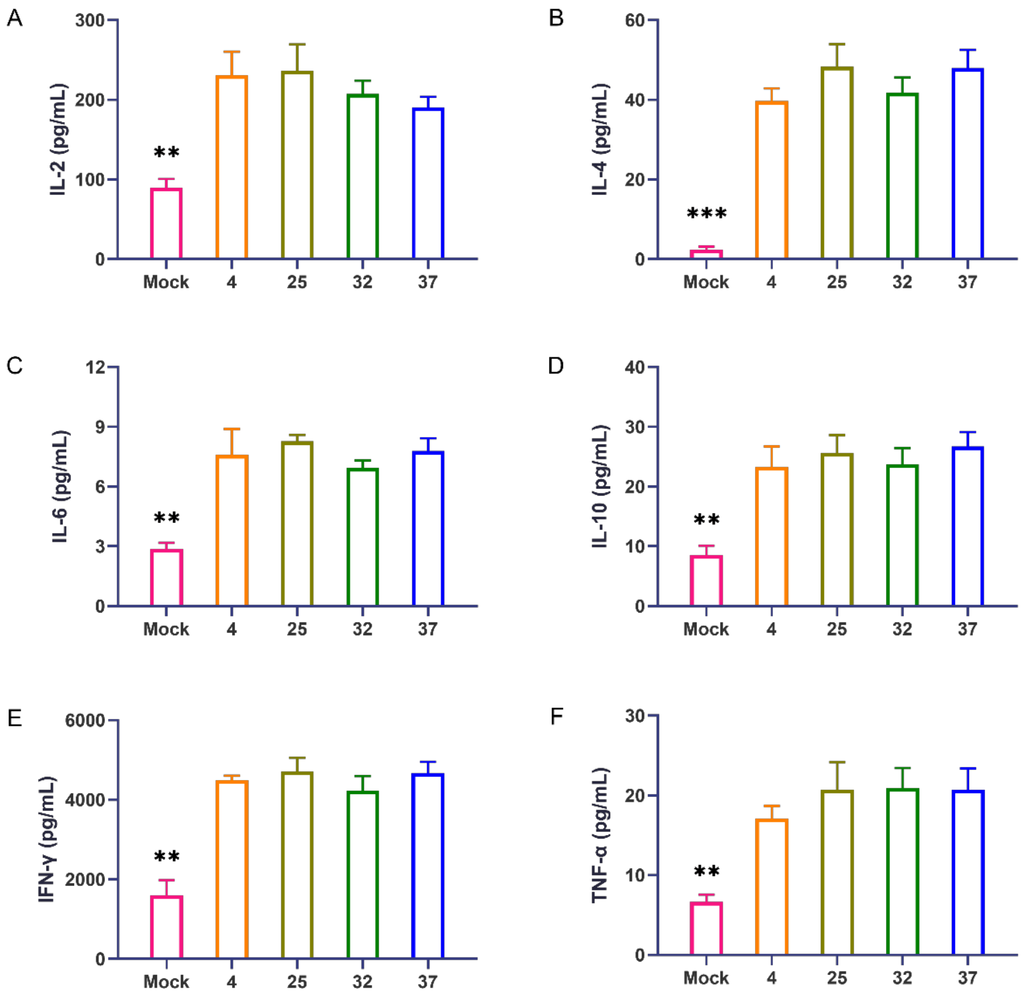

Supplement 1 Cytokine levels produced by CD19 CAR-T cells in the culture medium on day 6

(A-F) IL-2, IL-4, IL-6, IL-10, IFN-γ and TNFα levels in the culture medium on the 6th day of CD19 CAR-T cell production. Three independent experiments were conducted. Mean ± SD. **$p < 0.01$, ***$p < 0.001$.

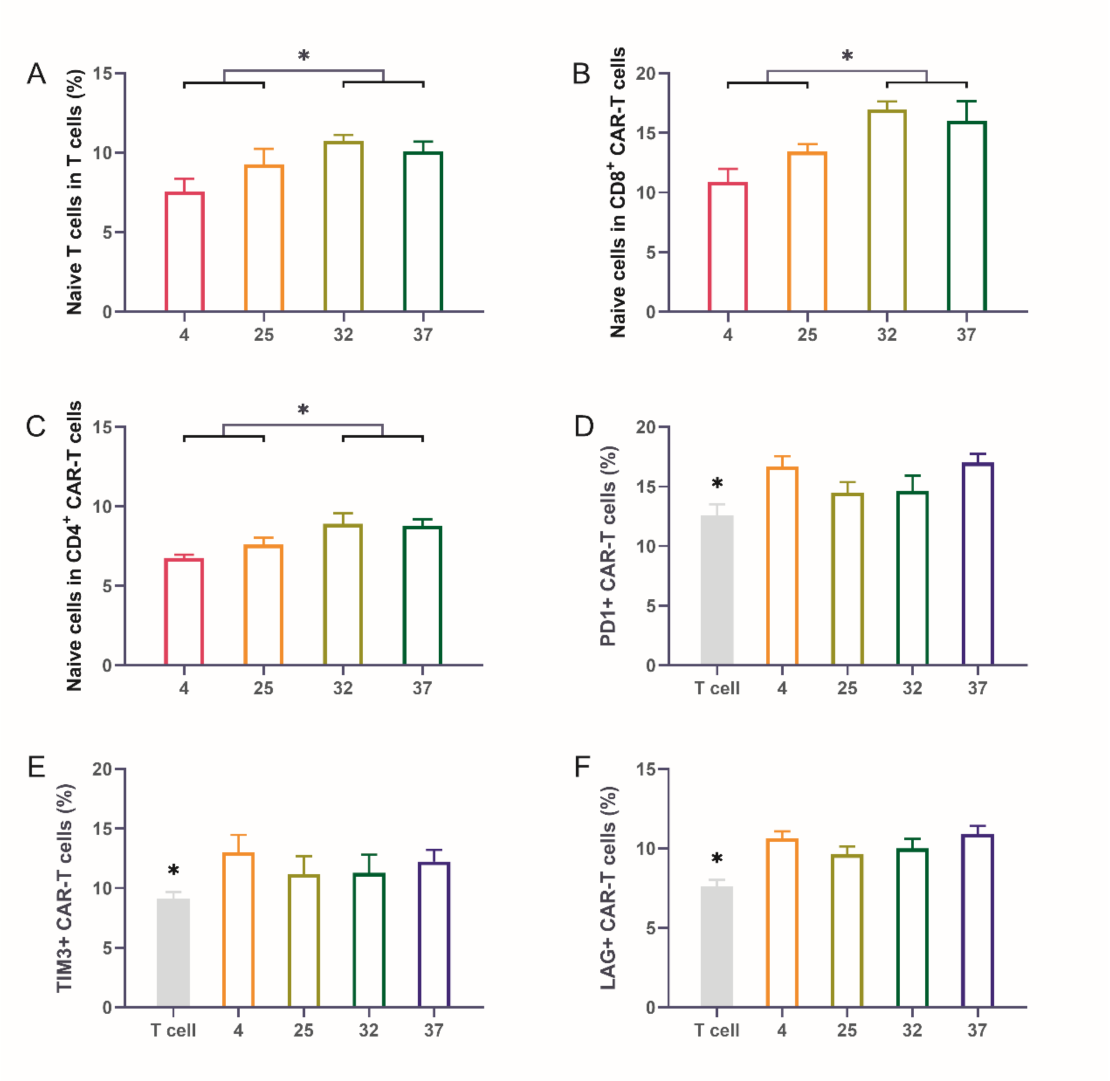

Supplement 2 Infection temperature can affect the distribution of CD123 CAR-T cell subsets and the expression of immune checkpoints

(A-C) The proportions of naive T cells among CD3-positive, CD8-positive and CD4-positive CD123 CAR-T cells generated at different infection temperatures. **(D-F)** The expression of PD1, TIM3, and LAG3 on the surface of CD123 CAR-T cells generated at different infection temperatures. Three independent experiments were conducted. Mean ± SD. *$p < 0.05$.

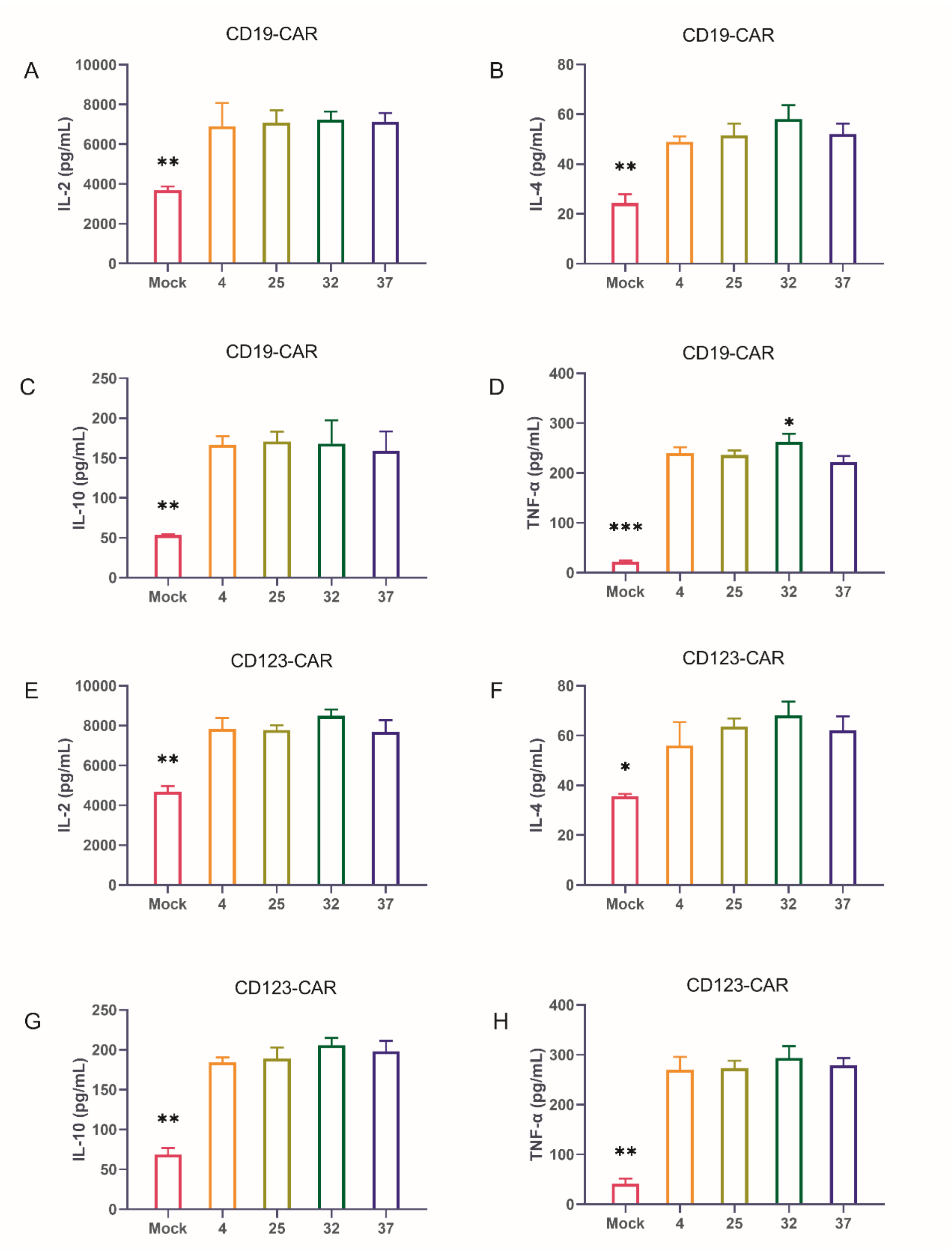

Supplement 3 Levels of other secreted cytokines after incubation of CD19 CAR-T and CD123 CAR-T cells with tumor cells

(A) IL-2 level after coincubation of CD19 CAR-T cells and tumor cells. **(B)** IL-2 level after coincubation of CD123 CAR-T cells and tumor cells. **(C)** IL-2 level after coincubation of CD19 CAR-T cells and tumor cells. **(D)** IL-4 level after coincubation of CD123 CAR-T cells and tumor cells. **(E)** IL-10 level after coincubation of CD19 CAR-T cells and tumor cells. **(F)** IL-4 level after coincubation of CD123 CAR-T cells and tumor cells. **(G)** TNFα levels after coincubation of CD19 CAR-T cells and tumor

cells. **(H)** TNFα levels after coincubation of CD123 CAR-T cells and tumor cells. Three independent experiments were conducted. Mean ± SD. *$p < 0.05$, **$p < 0.01$, ***$p < 0.001$.
